# The Long Noncoding RNA HOTAIR Contributes to Cisplatin Resistance of Human Lung Adenocarcinoma Cells via downregualtion of p21^WAF1/CIP1^ Expression

**DOI:** 10.1371/journal.pone.0077293

**Published:** 2013-10-14

**Authors:** Zhili Liu, Ming Sun, Kaihua Lu, Jing Liu, Meiling Zhang, Weiqin Wu, Wei De, Zhaoxia Wang, Rui Wang

**Affiliations:** 1 Department of Oncology, the Second Affiliated Hospital of Nanjing Medical University, Nanjing, P.R. China; 2 Department of Biochemistry and Molecular Biology, Nanjing Medical University, Nanjing, P.R. China; 3 Department of Oncology, the First Affiliated Hospital of Nanjing Medical University, Nanjing, P.R. China; 4 Department of Medical Oncology, Jinling Hospital, School of Medicine, Nanjing University, Nanjing, P.R. China; Cincinnati Children's Hospital Medical Center, United States of America

## Abstract

HOTAIR, a long intervening non-coding RNA (lincRNA), associates with the Polycomb Repressive Complex 2 (PRC2) and is reported to reprogram chromatin organization and promote tumor progression. However, little is known about the roles of this gene in the development of chemoresistance phenotype of lung adenocarcinoma (LAD). Thus, we investigated the involvement of HOTAIR in the resistance of LAD cells to cisplatin. In this study, we show that HOTAIR expression was significantly upregulated in cisplatin-resistant A549/DDP cells compared with in parental A549 cells. Knockdown of HOTAIR by RNA interference could resensitize the responses of A549/DDP cells to cisplatin both *in vitro* and *in vivo*. In contrast, overexpression of HOTAIR could decrease the sensitivity of A549 and SPC-A1 cells to cisplatin. We also found that the siRNA/HOTAIR1-mediated chemosensivity enhancement was associated with inhibition of cell proliferation, induction of G_0_/G_1_ cell-cycle arrest and apoptosis enhancement through regulation of p21^WAF1/CIP1^ (p21) expression. Also, pcDNA/p21or siRNA/p21 could mimic the effects of siRNA/HOTAIR1 or pcDNA/HOTAIR on the sensitivity of LAD cells to cisplatin. Importantly, siRNA/p21 or pcDNA/p21 could partially rescue the effects of siRNA/HOTAIR1 or pcDNA/HOTAIR on both p21 expression and cisplatin sensitivity in LAD cells. Further, HOTAIR was observed to be significantly downregulated in cisplatin-responding LAD tissues, and its expression was inversely correlated with p21 mRNA expression. Taken together, our findings suggest that upregulation of HOTAIR contributes to the cisplatin resistance of LAD cells, at least in part, through the regulation of p21 expression.

## Introduction

Lung cancer is the leading cause of cancer-related death worldwide [1]. Non-small cell lung cancer (NSCLC), the most common type of lung cancer, currently accounts for 70-80% of all lung cancer cases [2]. Lung adenocarcinoma (LAD), one histological subtype of NSCLC, has become the most common histologic type among all lung cancers diagnosed. Platinum based combination chemotherapy is the standard chemotherapy for NSCLC, and cisplatin, a member of the family of chemotherapy drugs known as platinum containing compounds or alkylating agents, remains the most widely employed first-line chemotherapeutic agent for the treatment of lung cancer [3]. However, individuals respond to chemotherapy differently and the efficacy of cisplatin treatment is often impaired by the emergence of chemoresistance. Thus, a better understanding the molecular mechanisms underlying the development of chemoresistance would promote our understanding of LAD development and treatment failure. 

Currently, the mechanisms underlying resistance development to chemotherapeutic agents are still not fully understood. Recently, evidence has been accumulating to suggest that a significant relationship between drug-resistant and epigenetic alterations exists [4-6]. MicroRNA (miRNAs) and long non-coding RNAs (lncRNAs) are the major regulatory noncoding RNAs that regulate gene expression at epigenetic, transcriptional, and post-transcriptional processing levels [7,8]. An increasing number of contemporary studies have shown that altered microRNA expression may play an important role in the chemoresistance of cancer cells by impairing cellular responses that affect cell cycle arrest, apoptosis, and DNA damage repair [9]. The increasing prevalence of miRNA-mediated drug resistance data has led to an increase in studies focused on targeting miRNAs as strategies for therapeutic intervention. Unfortunately, the correlations between lncRNAs and tumor chemoresistance are rarely reported, and need to be more clearly elucidated before these therapeutic strategies can be fully developed and undergo clinical assessment. HOTAIR is one of the few biologically well-documented lncRNAs, with a length of 2158 bp and a functional role in the mediation of trans-silencing [10]. HOTAIR has been found to be overexpressed in a variety of human cancers and determined to be a negative prognostic indicator in breast, colon, liver, and pancreatic cancer patient survival, evidencing a close association with increase in cancer cell metastasis [11-14]. Zhuang and his colleagues showed that tumor-promoting Col-1 up-regulates the expression of HOTAIR in NSCLC cells, suggesting that HOTAIR might play critical roles in lung tumorigenesis [15]. Although numerous studies have demonstrated the critical functions of HOTAIR in tumor development and metastasis, whether HOTAIR plays an import role during development of chemoresistance in human LADs is still unclear. 

In the present study, qRT-PCR assay was performed to detect the expression of HOTAIR in cisplatin-resistant A549/DDP, its parental A549 and another LAD cell line SPC-A1. Then, functional analyses of HOTAIR was conducted to evaluate the effects of HOTAIR expression on the chemosensitivity of LAD cells to cisplatin both *in vitro* and in vivo. Also, the effects of HOTAIR knockdown on cell apoptosis and cell cycle of LAD cells via regulation of p21 expression were further investigated. This study shows for the ﬁrst time that upregulation of HOTAIR can promote the resistance of human LAD cells to cisplatin, at least partially by downregulating p21. Taken together, this study explores the validity of HOTAIR as a valid therapeutic target for the reversal of cisplatin resistance in LAD patients 

## Methods and Materials

### Cell lines and cell culture

The cisplatin-resistant human LAD cell line (A549/DDP) and its parental cell line (A549), and another LAD cell line (SPC-A1) (obtained from Cancer Institute, Chinese Academy of Sciences) were cultured in RPMI-1640 medium (Gibco BRL, Grand Island, NY) supplemented with 10% fetal bovine serum, 100 U/mL penicillin, and 100 μg/mL streptomycin. The CDDP-resistant A549 cell line was selected by continuous exposure to increasing concentrations of cisplain (CDDP). CDDP was added into exponentially growing cultures of A549 cells at a concentration of 0.005 μg/L and allowed to remain in the culture until cell growth resumed. The cultures were then split and treated again with progressively higher concentrations of CDDP. Over the course of selection, the docetaxel concentration was increased to 1.0μg/ml. The resulting subline was designated as A549/DDP cell line, which was cultured in medium containing 1.0 µg/ml CDDP. All cell lines were cultured under the atmosphere of 5% CO_2_ with humidity at 37°C. In all experiments, exponentially growing cells were used.

### Patients and tissue samples

A total of 41 tumor tissues were collected from advanced LAD patients who received cisplatin-based chemotherapy at the First or Second Affiliated Hospital of Nanjing Medical University during April 2007 and November 2009. All of the following criteria were met: patients who suffered from primary LAD; a histological diagnosis of LAD with at least one measurable lesion; a clinical stage of IIIB to IV; ﬁrst-line chemotherapy with cisplatin 25 mg/m^2^ on days 1, 2, 3 and gemcitabine 1000 mg/m^2^ on days 1, 8 or paclitaxel 80 mg/m^2^ on days 1, 8 every 21 days for a maximum of 4 cycles. Tissue samples were divided into ‘‘sensitive’’ (complete or partial response) and ‘‘insensitive’’ (stable or progressive disease) groups according to the patient’s responses assessed by medical image analysis and detection of serum tumor markers after 4 cycles of the cisplatin-based chemotherapy. Tumor staging was determined according to the sixth edition of the tumor-node-metastasis (TNM) classification of the International Union against Cancer. All patients or their guardians provided written informed consent, and the Chinese Medical Association Society of Medicine’s Ethics Committee approved all aspects of this study in accordance with the Helsinki Declaration.

### Ethics statement

The study was approved by the Ethic Committee of Nanjing University and it was performed in compliance with the Helsinki Declaration. Written informed consent was obtained for all patient samples. All experimental animals were housed under specific pathogen-free conditions. All experimental procedures were approved by the Institutional Review Board of the Nanjing University. All procedures were performed in accordance with the Nanjing University Guide for the Care and Use of Laboratory Animals formulated by the National Society for Medical Research.

### Immunohistochemistry

Transplanted tumor tissues were immunostained for p21 protein. The signal was amplified and visualized using 3, 30-diaminobenzidine chromogen followed by counterstaining with hematoxylin. Expression was considered positive when 50% or more of cancer cells were stained. Anti-p21 (1:50) or Anti-PCNA (1:100) was purchased from Cell Signaling Technology (MA, USA).

### Construction of plasmid vector

To ectopically express HOTAIR and p21, the HOTAIR and p21 gene was subcloned into pcDNA3.1(+) (Invitrogen, USA) by PCR method using the following primers: HOTAIR, sense, 5’-CATGGATCCACATTCTGCCCTGATTTCCGGAACC-3’; reverse, 5’-*ACTCTCGAGCCACCAC-*

*ACACACACAACCTACAC*-3’. p21, sense, 5’-CACCATGTCAGAACCGGCTGGGGATG-3’; reverse, 5’-TTAGGGCTTCCTCTTGGAGAAGATCAGC-3’. The PCR products were resolved by electrophoresis on a 1% agarose gel and the gel was stained with ethidium bromide and imaged. The identity of the PCR product was confirmed by direct sequence analysis. The open reading frame of p21 that was generated by PCR was then inserted into the pcDNA 3.1 expression vector which was named pcDNA/p21. The recombinant vector was confirmed by the digestion analysis of restriction endonuclease and DNA sequencing.

### Transfection of siRNAs and plasmid vectors

The cells were seeded into 6-well plates and transfected with 50 nM siRNAs targeting HOTAIR (siRNA/HOTAIR1: 5’-UUUUCUACCAGGUCGGUAC-3’; siRNA/HOTAIR2: 5’- *AAUUCUUA-*

*AAUUGGGCUGG*-3’) (GenePharma, Shanghai, China) or siRNA/p21 (sc-29428) (Santa cruz biotechnology, USA) or siRNA/control (5’-CUACAACAGCCACAACGUCdTdT-3’) using Lipofectamine 2000 (Invitrogen, USA) according to the instructions provided by the manufacturer. The stable p21 or HOTAIR-expressing cells were generated by transfection with either pcDNA/p21, pcDNA/HOTAIR or pcDNA/control vectors using Lipofectamine 2000, followed by selection with G418.

### RNA extraction and quantitative real-time PCR

Total RNA was extracted from tissues or cell lines using the TRIzol reagent (Invitrogen, USA). For qRT-PCR assay, RNA was reverse transcribed to cDNA from 1.0 ug of total RNA using a Reverse Transcription Kit (Takara, Shiga, Japan). Real-time PCR (RT-PCR) analyses were conducted using the Power SYBR Green (Takara, Shiga, Japan). All protocols were carried out according to the instructions provided by the manufacturer. HOTAIR expression was determined by qRT-PCR using the following primer sequences: sense, 5’-CAGTGGGGAACTCTGACTCG-3’; and reverse, 5’-GTGCCTGGTGCTCTCTTACC-3’. Glyceraldehyde-3-phosphate dehydrogenase (GAPDH) was used as an internal control and the primers were as follows: sense, 5’-GTCAACGGATTTGGTCTGTATT-3’; and reverse, 5’-AGTCTTCTGGGTGGCAGTGAT-3’. All qRT-PCR assays were performed on an ABI 7500 Fast Real-Time PCR System (Applied Biosystems, CA, USA). 

### Western blot assay

The cells were lysed using the mammalian protein extraction reagent RIPA (Beyotime, Beijing, China) supplemented with a protease inhibitor cocktail (Roche, CA, USA) and phenylmethylsulfonyl ﬂuoride (PMSF) (Roche, CA, USA). Approximately a 50 µg protein extraction was separated by 10% SDS-PAGE, transferred to 0.22 mm nitrocellulose (NC) membrane (Sigma), and incubated with speciﬁc antibodies. Autoradiograms were quantiﬁed by densitometry using Quantity One software (Bio-Rad, CA, USA). GAPDH antibody was used as a control, and rabbit anti-p21, Bax, Bim, Bcl-xl, and Bcl-2 were provided by Cell Signaling Technology (MA, USA).

### Flow cytometric analysis of cell cycle or apoptosis

Double staining with ﬂuorescein isothiocyanate (FITC)-Annexin V and propidium iodide was completed using a FITC Annexin V Apoptosis Detection Kit (BD Biosciences, Shanghai, China) according to the instructions provided by the manufacturer. Cells were analyzed using a flow cytometer (FACScan; BD Biosciences, Shanghai, China) equipped with Cell Quest software (BD Biosciences, Shanghai, China), resulting in classification of cells as viable cells, dead cells, early apoptotic cells, and apoptotic cells. The relative ratio of early apoptotic cells was compared with the control transfectant in each experiment. Cells for cell-cycle analysis were stained with propidium oxide using the BD Cycletest Plus DNA Reagent Kit (BD Biosciences, Shanghai, China) following the protocol provided by the manufacturer. Analysis was conducted FAC Scan (BD Biosciences, Shanghai, China). The percentage of cells in G_0_/G_1_, S or G_2_/M phase was counted and compared. Each experiment was performed at least in triplicate.

### In vitro chemosensitivity assay

The *in vitro* chemosensitivity of cisplatin-resistant or parental A549 cells to cisplatin was determined by 2.7.3-(4,5-dimethylthiazol-2-yl)-2,5-diphenyltetrazolium bromide (MTT) assay. Briefly, cells were seeded into 96-well plates (3.5×10^3^ cells/well) and allowed to attach overnight. After cellular adhesion was achieved, cells were treated with various concentrations (0, 1, 5, 10, 12, 16, 18, 20, 22 and 24 μg/ml) of cisplatin. At 0, 24, 48, 72, and 96 h, cell vitality was assessed using 0.5 mg/mL MTT (Sigma, MO, USA) solution. Approximately 4 h later, the medium was replaced with 150 µl dimethyl sulfoxide (DMSO, Sigma, MO, USA) and vortexed for 10 min. The absorbance at 490 nm (A490) of each well was read using a spectrophotometer. Each experiment was performed at least in triplicate.

### In vivo chemosensitivity assay

The male athymic BALB/c nude mice aged 5 weeks were maintained under speciﬁc pathogen-free conditions and manipulated according to protocols approved by the Shanghai Medical Experimental Animal Care Commission. Tumor cells were transiently transfected with siHOTAIR1 or siRNA/control and harvested from 6-well cell culture plates, washed with PBS, and resuspended at a concentration of 2.0×10^7^ cells/mL. A volume of 0.1 ml of suspended cells was subcutaneously injected into a single side of the posterior flank of each mouse. Tumor growth was examined every other day, and tumor volumes were calculated using the equation *V* = 0.4 × *D* × d^2^ (*V*, volume; *D*, longitudinal diameter; *d*, latitudinal diameter). When the average tumor size reached approximately 50 mm^3^, cisplatin was administered by intraperitoneal injection with a dose of 3 mg/kg, once every other day, for a total of 3 doses. At 4 weeks after injection, mice were killed, and the subcutaneous growth of each tumor was examined. The primary tumors were excised, paraffin-embedded, formalin-fixed, and performed H&E staining, immunostaining analysis for p21 or PCNA (proliferating cell nuclear antigen) protein expression and analyzed the apoptosis by TUNEL Apoptosis Detection Kit (KeyGEN, China) according to the manufacturer’s instructions.

### Statistical analysis

All data are presented as means±SE and were analyzed using Prism 5.0 software (GraphPad). A student’s *t*-test (2-tailed), one-way ANOVA, and Mann-Whitney U test were conducted to analyze the *in vitro* and *in vivo* data using SPSS 16.0 software (IBM, IL, USA). *P*-values of less than 0.05 were considered significant (*P*<0.05).

## Results

### HOTAIR is significantly upregulated in cisplatin-resistant human LAD cell line (A549/DDP) compared with parental LAD cell line (A549)

**Figure 1:**
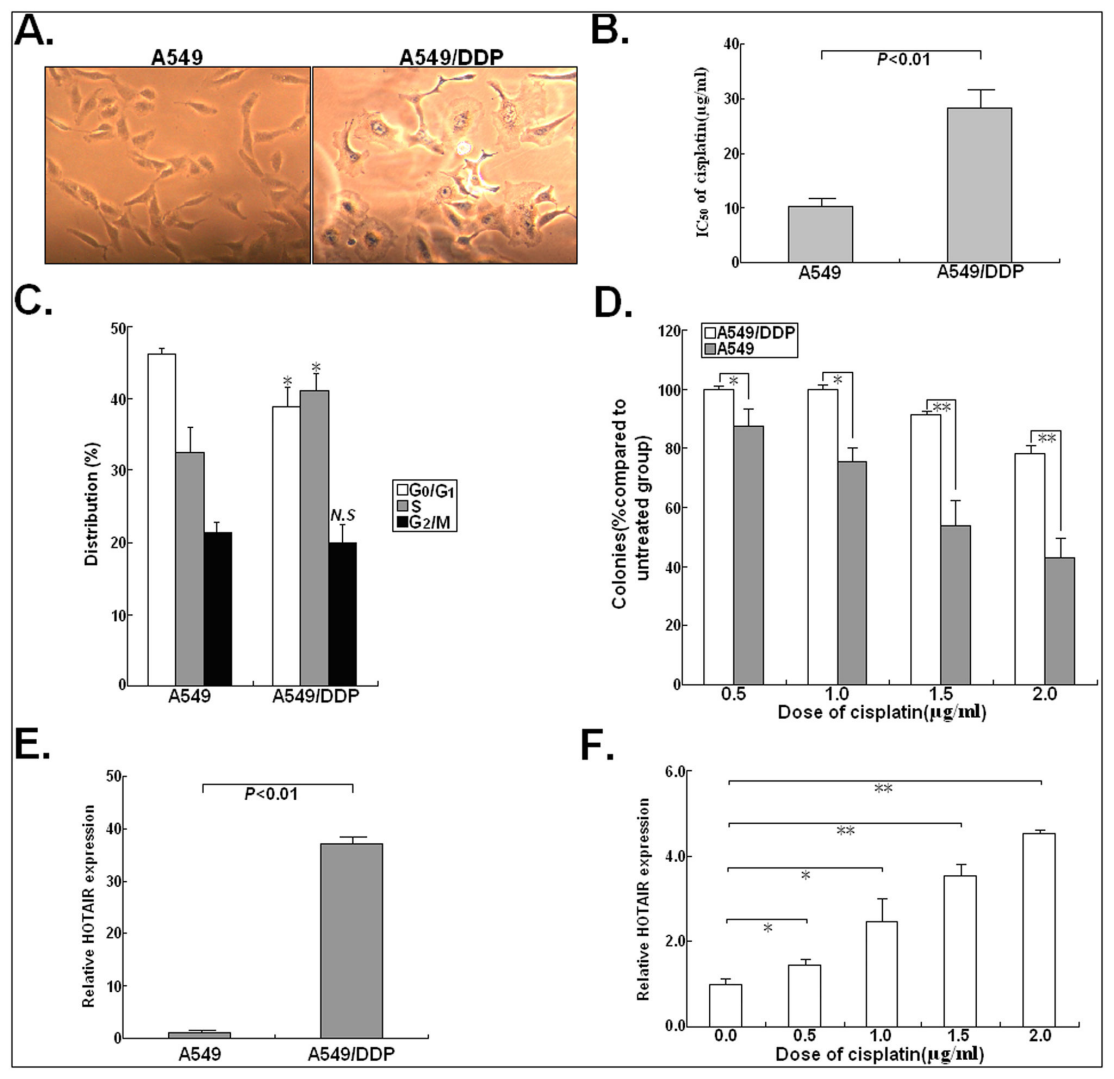
Expression of HOTAIR in cisplatin-resistant A549/DDP cells is significantly upregulated compared with that in parental A549 cells. (**A**) Morphologies of A549 and A549/DDP cells. Cells were grown to 70% confluency and then photographe under 40× magnification. (**B**) The IC_50_ value of cisplatin to A549/DDP cells was significantly higher than that to A549 cells. (**C**) Flow cytometric analysis of cell cycle distribution in A549 and A549/DDP cells. (**D**) The colony formation of A549 and A549/DDP cells treated with various concentrations of cisplatin (0.5, 1.0, 1.5 and 2.0 μg/L). (**E**) qRT-PCR analysis of HOTAIR expression in A549/DDP and A549 cells. (**F**) A549 cells were cultured in the presence of various concentrations of cisplatin (0.0, 0.5, 1.0, 1.5 or 2.0 μg/L) for 24h. qRT-PCR assay was performed to detect HOTAIR expression. GAPDH was used as an internal control. Results represent the average of three independent experiments (mean±SD). *N.S* indicates *P*>0.05 and * or ** indicates *P*<0.05 or <0.01, respectively.

The cisplatin-resistant LAD cell line (A549/DDP) was developed from parental A549 cell line. As observed by optical microscopy, A549/DDP cell line appears large swellings or spindle-shaped cell form compared with parental A548 cell line (Figure 1A). MTT assay was performed to detect the IC_50_ of cisplatin to A549/DDP and parental A549 cell line, and we showed that the IC_50_ of cisplatin to A549 cells and A549/CDDP cells was (10.35±1.28) μg/ml and (28.26±2.12) μg/ml, respectively (*P*<0.01; Figure 1B), suggesting that A549/CDDP cell line was 2.73 times more resistant than the parental A549 cell line. Next, flow cytometry was used to detect cell cycle distribution of A549/DDP and A549 cells, and showed that the percentage of G_0_/G_1_ phase cells was decreased and the percentage of S phase cells was increased in resistant A549/DDP cells relative to parental A549 cells (*P*<0.05; Figure 1C). Colony formation assays also showed significant cisplatin resistance in the A549/DDP compared with A549 cell line (Figure 1D). To further testify whether HOTAIR plays a critical role in the acquired cisplatin resistance of LAD cells, qRT-PCR assay was performed to detect the expression of HOTAIR in A549/DDP and parental A549 cells. Compared with that in parental A549 cells, the relative level of HOTAIR expression in A549/DDP cells was significantly increased by approximately 36.2 folds (*P*<0.01; Figure 1E). When parental A549 cells were treated with various concentrations of cisplatin (0.0, 0.5, 1.0, 1.5 and 2.0 μg/ml) for 24h, qRT-PCR assay showed that the relative level of HOTAIR expression was significantly increased (Figure 1F). Thus, a increasing expression level of HOTAIR in LAD cells responds to cisplatin treatment.

### Involvement of HOTAIR in cisplatin resistance in human LAD cells

**Figure 2:**
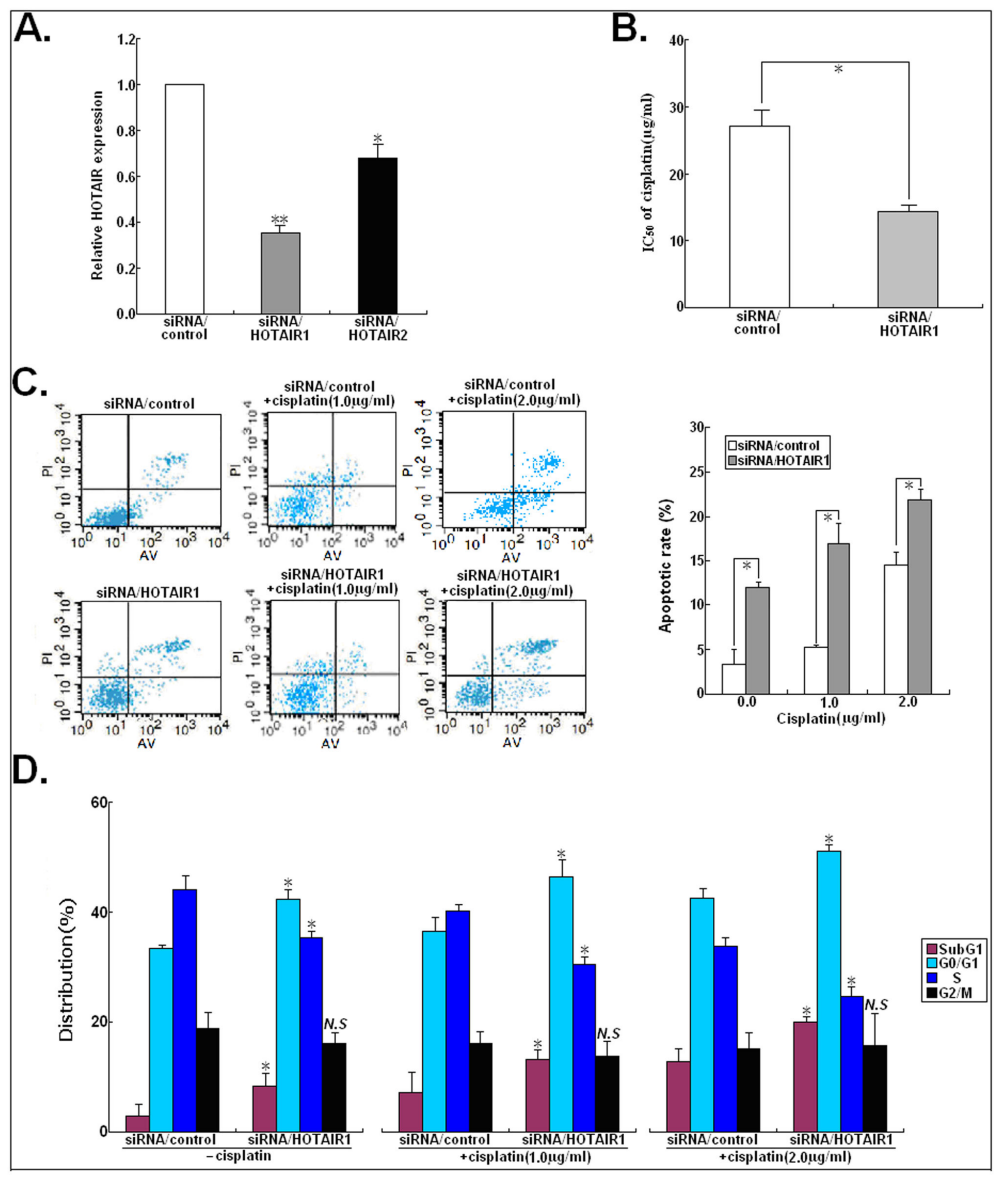
siRNA/HOTAIR significantly increases the in vitro sensitivity of A549/DDP to cisplatin. (**A**) 48h after A549/DDP cells transfected with siRNA/control, siRNA/HOTAIR1 or siRNA/HOTAIR2, qRT-PCR detection of HOTAIR expression in those cells. GAPDH was used as an internal control. (**B**) MTT analysis of the IC_50_ values of cisplatin to siRNA/HOTAIR1 or siRNA/control-transfected A549/DDP cells. (**C**) Flow cytometry analysis of apoptosis in siRNA/control or siRNA/HOTAIR1-transfected A549/DDP cells combined with various concentrations of cisplatin (0.0, 1.0 or 2.0 μg/L). (**D**) Flow cytometry analysis of cell cycle distribution in siRNA/control or siRNA/HOTAIR1-transfected A549/DDP cells combined with various concentrations of cisplatin (0.0, 1.0 or 2.0 μg/L). Results represent the average of three independent experiments (mean±SD). *N.S* indicates *P*>0.05 and * or ** indicates *P*<0.05 or <0.01, respectively.

To further investigate the effect of HOTAIR expression on the sensitivity of LAD cells to cisplatin, resistant A549/DDP cells were transfected with siRNA/control, siRNA/HOTAIR1 or siRNA/HOTAIR2, respectively. 48h after transfection, qRT-PCR assay was performed to detect the expression of HOTAIR (Figure 2A). Compared with siRNA/control-transfected cells, the expression of HOTAIR was significantly decreased by about 64.5% in siRNA/HOTAIR1-transfected A549/DDP cells (*P*<0.01). However, siRNA/HOTAIR2 could slightly inhibit the expression of HOTAIR. Thus, siRNA/HOTAIR1 was used in the following experiments. Next, MTT assay was performed to detect the effects of HOTAIR expression on the IC_50_ of cisplatin to A549/DDP cells, and results showed that siRNA/HOTAIR1 could significantly decrease the IC_50_ of cisplatin to A549/DDP cells by approximately 47.12% (*P*<0.05; Figure 2B). Since siRNA/HOTAIR1 could significantly increase the chemosensitivity of A549/DDP cells to cisplatin, we further investigated its roles and mechanisms in cisplatin resistance. When A549/DDP cells was transfected with siRNA/HOTAIR1 combined with cisplatin treatment (0.0, 1.0 and 2.0 μg/ml), it was found that siRNA-mediated HOTAIR downregulation could significantly increase cisplatin-induced apoptosis of cisplatin-resistant LAD cells (*P*<0.05; Figure 2C). Compared with those siRNA/control-transfected A549/DDP cells, the percent of siRNA/HOTAIR1-transfected A549/DDP cells in subG1 and G_0_/G_1_ phase of cell cycle increased gradually and the percentage of cells in S phase decreased gradually with increasing doses of cisplatin (*P*<0.05) (Figure 2D). Thus, downregulation of HOTAIR could reverse the cisplatin resistance of A549/DDP cells by inducing apoptosis enhancement and G_0_/G_1_ cell cycle arrest. 

**Figure 3:**
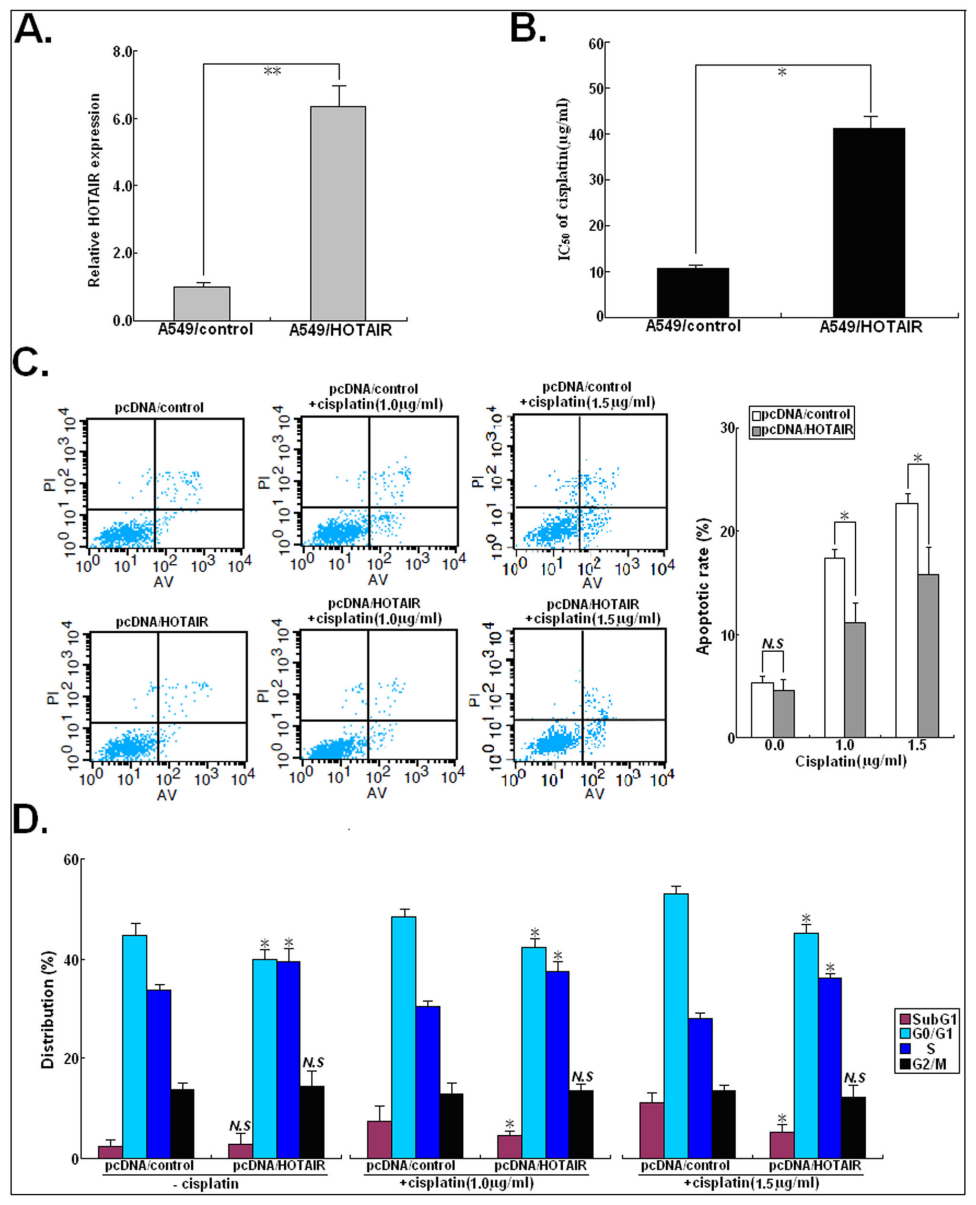
pcDNA/HOTAIR significantly promotes the resistance of parental A549 cells to cisplatin. (**A**) qRT-PCR detection of HOTAIR expression in A549 cells stably transfected with pcDNA/control or pcDNA/HOTAIR. GAPDH was used as an internal control. (**B**) MTT analysis of the IC_50_ values of cisplatin to A549/control or A549/HOTAIR cells. (**C**) Flow cytometry analysis of apoptosis in A549/control or A549/HOTAIR cells combined with various concentrations of cisplatin (0.0, 1.0 or 1.5 μg/L). (**D**) Flow cytometry analysis of cell cycle distribution in A549/control or A549/HOTAIR cells combined with various concentrations of cisplatin (0.0, 1.0 or 1.5 μg/L). Results represent the average of three independent experiments (mean±SD). *N.S* indicates *P*>0.05 and * or ** indicates *P*<0.05 or <0.01, respectively.

To further testify the roles of HOTAIR overexpression in the development of cisplatin resistance of LAD cells, pcDNA/HOTAIR was stably transfected into parental A549 cells. Compared with A549/control cells, the level of HOTAIR expression in A549/HOTAIR cells was significantly increased by about 534% (*P*<0.01; Figure 3A). Also, it was observed that upregulation of HOTAIR could significantly increase the IC_50_ of cisplatin to A549 cells by about 4.12 folds (*P*<0.05; Figure 3B). When A549 cells was transfected with pcDNA/HOTAIR combined with cisplatin treatment (0.0, 1.0 and 1.5 μg/ml), it was found that upregulation of HOTAIR could lead to the decreased cisplatin-induced apoptosis of parental A549 cells (*P*<0.05; Figure 3C). Also,  the percentage of pcDNA/HOTAIR-transfected A549 cells in subG1 and G_0_/G_1_ phases of cell cycle decreased gradually and the percentage of cells in S phase increased gradually with increasing doses of cisplatin (*P*<0.05; Figure 3D). To testify the above data, pcDNA/HOTAIR was stably transfected into another LAD cell line (SPC-A1), and the level of HOTAIR expression was increased by 436.8% in SPC-A1/HOTAIR (*P*<0.01; Figure S1A). Upregulation of HOTAIR could also significantly increase the IC_50_ of cisplatin to SPC-A1 cells by about 3.42 folds (*P*<0.05; Figure S1B). Likewise, upregulation of HOTAIR could induce the decreased cisplatin-induced apoptosis in SPC-A1 cells (Figure S1C). Cell cycle analyses indicated that upregulation of HOTAIR could induce the decreased percentage of cells in subG1 and G_0_/G_1_ phases of cell cycle and the increased percentage of cells in S phase with increasing doses of cisplatin (Figure S1D). Thus, upregulation of HOTAIR might decrease the sensitivity of parental LAD cells to cisplatin by reducing apoptosis and the percentage of cells in G0/G1 phase of cell cycle.

### p21 mediates HOTAIR-stimulated cisplatin resistance in LAD cells

**Figure 4:**
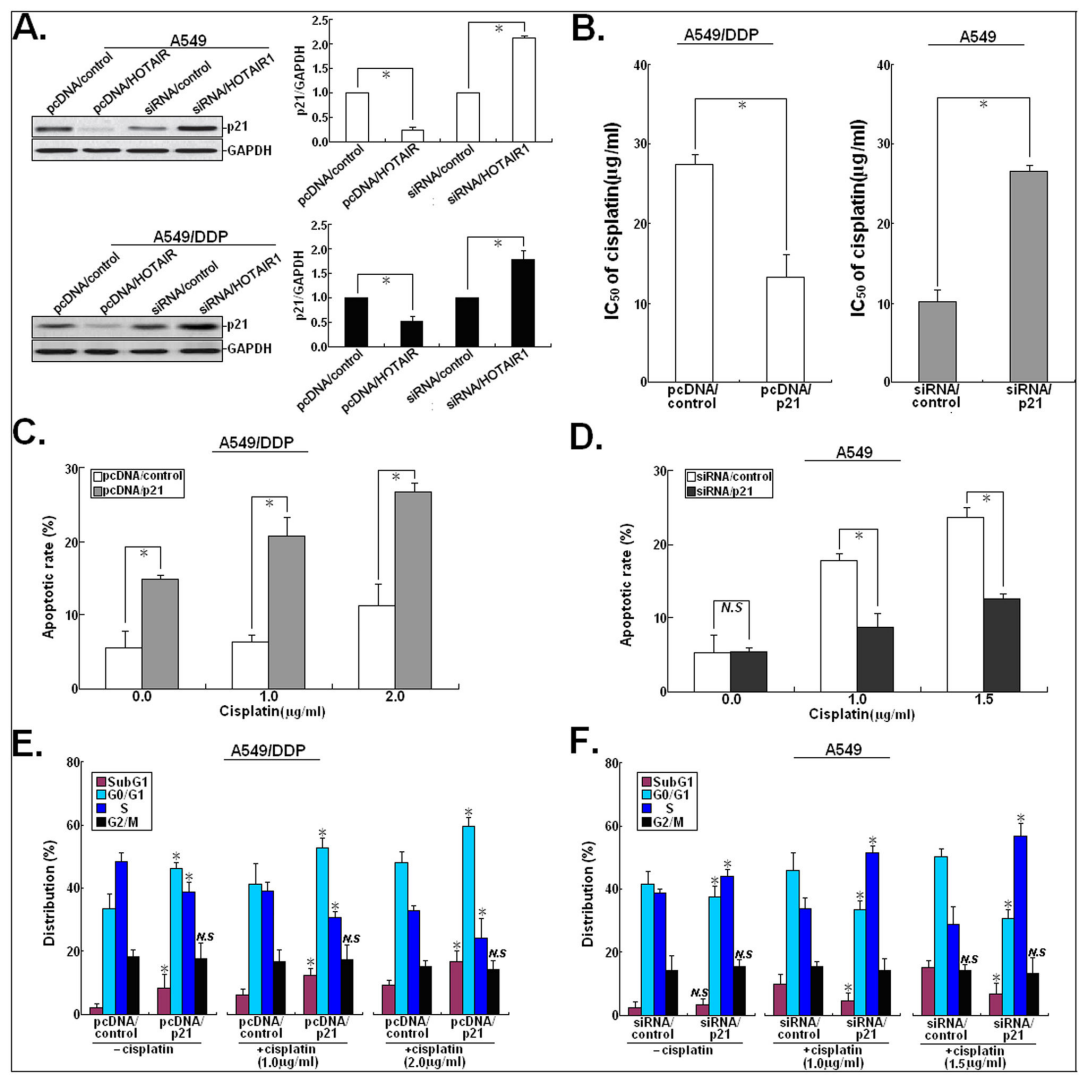
pcDNA/p21 or siRNA/p21 could mimic the effects of siRNA/HOTAIR1 or pcDNA/HOTAIR on the sensitivity of LAD cells to cisplatin. (**A**) Western blot analysis of the effect of HOTAIR on p21 protein expression in pcDNA/HOTAIR (or pcDNA/control) or siRNA/HOTAIR1 (or siRNA/control)-transfected A549 or A549/DDP cells. GAPDH was used as an internal control. (**B**) MTT analysis of the IC_50_ values of cisplatin to pcDNA/control or pcDNA/p21-transfected A549/DDP cells and siRNA/control or siRNA/p21-transfected A549 cells. (**C**) Flow cytometry analysis of apoptosis in pcDNA/control or pcDNA/p21-transfected A549/DDP cells combined with various concentrations of cisplatin (0.0, 1.0 or 2.0 μg/L). (**D**) Flow cytometry analysis of apoptosis in siRNA/control or siRNA/p21-transfected A549 cells combined with various concentrations of cisplatin (0.0, 1.0 or 1.5 μg/L). (**E**) Flow cytometry analysis of cell cycle distribution in pcDNA/control or pcDNA/p21-transfected A549/DDP cells combined with various concentrations of cisplatin (0.0, 1.0 or 2.0 μg/L). (**F**) Flow cytometry analysis of cell cycle distribution in siRNA/control or siRNA/p21-transfected A549 cells combined with various concentrations of cisplatin (0.0, 1.0 or 1.5 μg/L). Results represent the average of three independent experiments (mean±SD). *N.S* indicates *P*>0.05 and * or ** indicates *P*<0.05 or <0.01, respectively.

Then, we want to investigate whether HOTAIR could affect the chemosensitivity of LAD cells by silencing multiple tumor suppressors. HOTAIR can trimethylate histone H3 lysine-27 (H3K27me3) of the HOXD locus with the polycomb-repressive complex 2 (PRC2), which is composed of EZH2, SUZ12, and EED [16]. Previous studies have shown that EZH2 and H3K27me3 are both enriched in the promoter region of p21 [17]. Thus, Western blot assay was first performed to detect the effect of HOTAIR expression on p21 protein. As shown in Figure 4A, upregulation of HOTAIR could lead to the decreased p21 protein expression in parental A549 and A549/DDP cells and downregulation of HOTAIR could also lead to the increased p21 protein expression in both cells (*P*<0.05). Likewise, we also found that upregulation or downregulation of HOTAIR could induce the same effects on the expression of p21 protein in another LAD cell line (SPCA-1) (Figure S2A). Then, the effect of p21 expression on the sensitivity of LAD cells to cisplatin was determined. After pcDNA/p21 or siRNA/p21 was transiently or stably transfected into A549/DDP or A549 cells, the IC_50_ values of cisplatin to those cells was determined (Figure 4B). Upregulation of p21 could significantly decrease the IC_50_ value of cisplatin to A549/DDP cells by about 51.5% (*P*<0.05), while downregulation of p21 could increase the IC_50_ value of cisplatin to A549 cells by about 154.8% (*P*<0.05). In addition, we analyzed the effect of p21 expression on the cisplatin-induced apoptosis and cell cycle in A549/DDP or A549 cells. Upregulation of p21 could enhance the cisplatin-induced apoptosis in A549/DDP cells (*P*<0.05; Figure 4C), while downregulation of p21 could reduce the cisplatin-induced apoptosis in A549 cells (*P*<0.05; Figure 4D). Meanwhile, we found that the percentage of pcDNA/p21-transfected A549/DDP cells in subG1 and G_0_/G_1_ phases of cell cycle increased and the percentage of cells in S phase decreased with increasing doses of cisplatin (Figure 4E). Also, the percentage of siRNA/p21-transfected A549 cells in subG1 and G_0_/G_1_ phases of cell cycle decreased gradually and the percentage of cells in S phase increased gradually with increasing doses of cisplatin (Figure 4F). These data suggested that upregulation of p21 could mimic the effect of siRNA/HOTAIR on the cisplatin sensitivity of A549/DDP cells and downregulation of p21 could also mimic the effect of pcDNA/HOTAIR on the cisplation sensitivity of A549 cells. 

**Figure 5:**
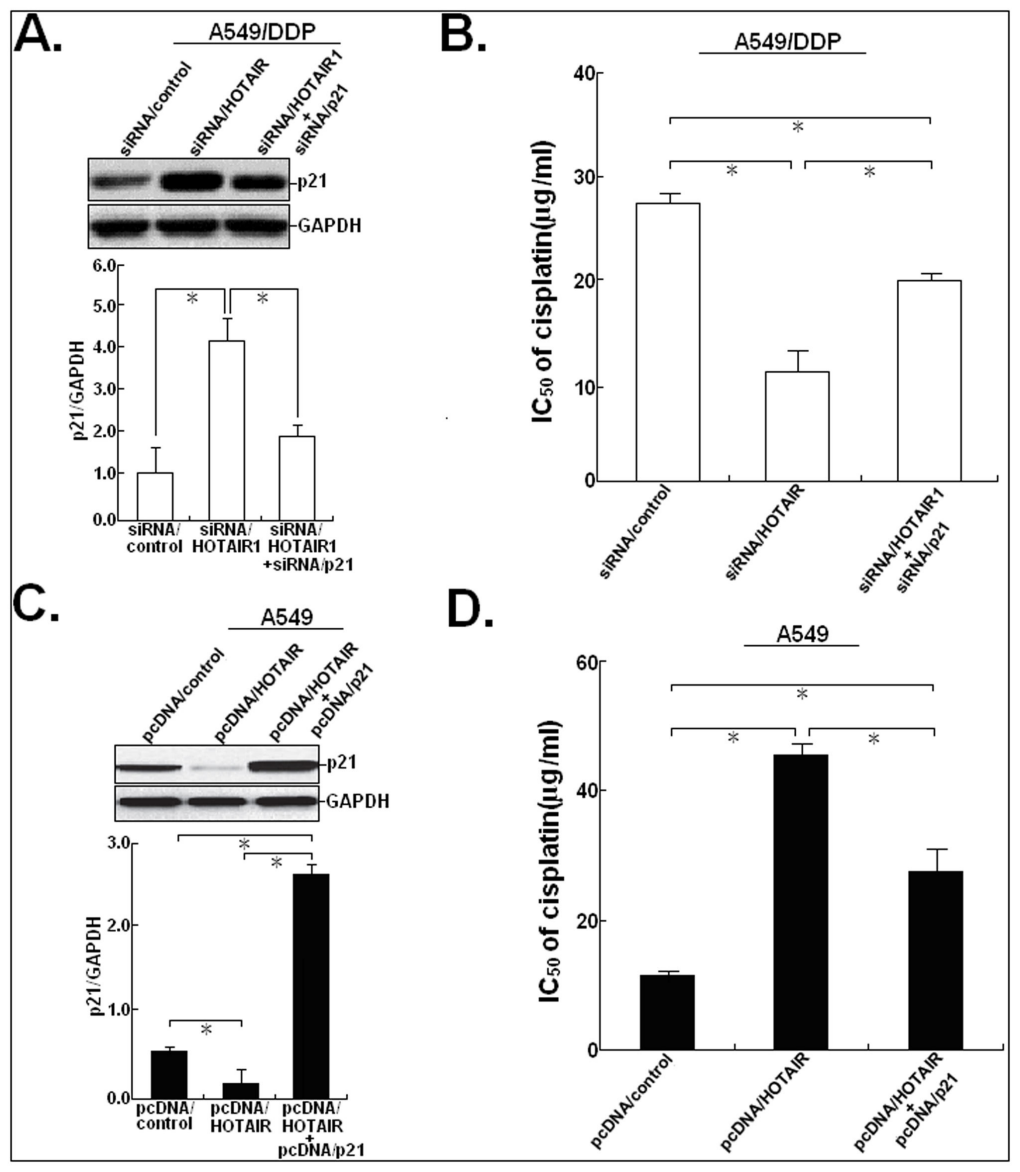
siRNA/p21 or pcDNA/21 reverses the effects of siRNA/HOTAIR1 or pcDNA/HOTAIR on the chemosensitivity of LAD cells to cisplatin. (**A**) 48h after A549/DDP cells transfected with siRNA/control, siRNA/HOTAIR1 alone or combination with siRNA/p21, Western blot detection of p21 protein expression in those cells. GAPDH was used as an internal control. (**B**) MTT analysis of the IC_50_ values of cisplatin to A549/DDP cells transfected with siRNA/control, siRNA/HOTAIR1 alone or combination with siRNA/p21. (**C**) 48h after A549 cells transfected with pcDNA/control, pcDNA/HOTAIR alone or combination with pcDNA/p21, Western blot detection of p21 protein expression in those cells. GAPDH was used as an internal control. (**D**) MTT analysis of the IC_50_ values of cisplatin to A549/DDP cells transfected with pcDNA/control, pcDNA/HOTAIR alone or combination with pcDNA/p21. Results represent the average of three independent experiments (mean±SD). *N.S* indicates *P*>0.05 and * or ** indicates *P*<0.05 or <0.01, respectively.

To further explore whether p21 play critical roles in HOTAIR-mediated cisplatin resistance in LAD cells, A549/DDP cells were co-transfected with siRNA/HOTAIR and siRNA/p21. 48h after transfection, Western blot assay was performed to detect the expression of p21 protein. It was observed that siRNA/p21 could rescue the increased expression of p21 protein in A549/DDP cells induced by siRNA/HOTAIR (*P*<0.05; Figure 5A). Also, siRNA/p21 could partially rescue the decreased IC_50_ value of cisplatin to A549/DDP cells induced by siRNA/HOTAIR (*P*<0.05; Figure 5B). Then, the stably transfected A549/control or A549/HOTAIR cells were transfected with pcDNA/p21 vector. 48h after transfection, Western blot assay showed that siRNA/p21 could rescue the decreased p21 protein expression in A549cells induced by pcDNA/HOTAIR (*P*<0.01; Figure 5C). Likewise, pcDNA/p21 could rescue the increased IC_50_ value of cisplatin to A549 cells induced by pcDNA/HOTAIR (*P*<0.01; Figure 5D). Moreover, the decreased p21 protein expression and the increased IC_50_ value of cisplatin in SPC-A1 cells induced by upregulation of HOTAIR could also be rescued by upregulation of p21 (Figure S2B and C). These data further suggested that p21 might be an important mediator in HOTAIR-induced cisplatin resistance of LAD cells. 

### HOTAIR promotes the in vivo resistance of LAD cells to cisplatin by targeting p21

**Figure 6:**
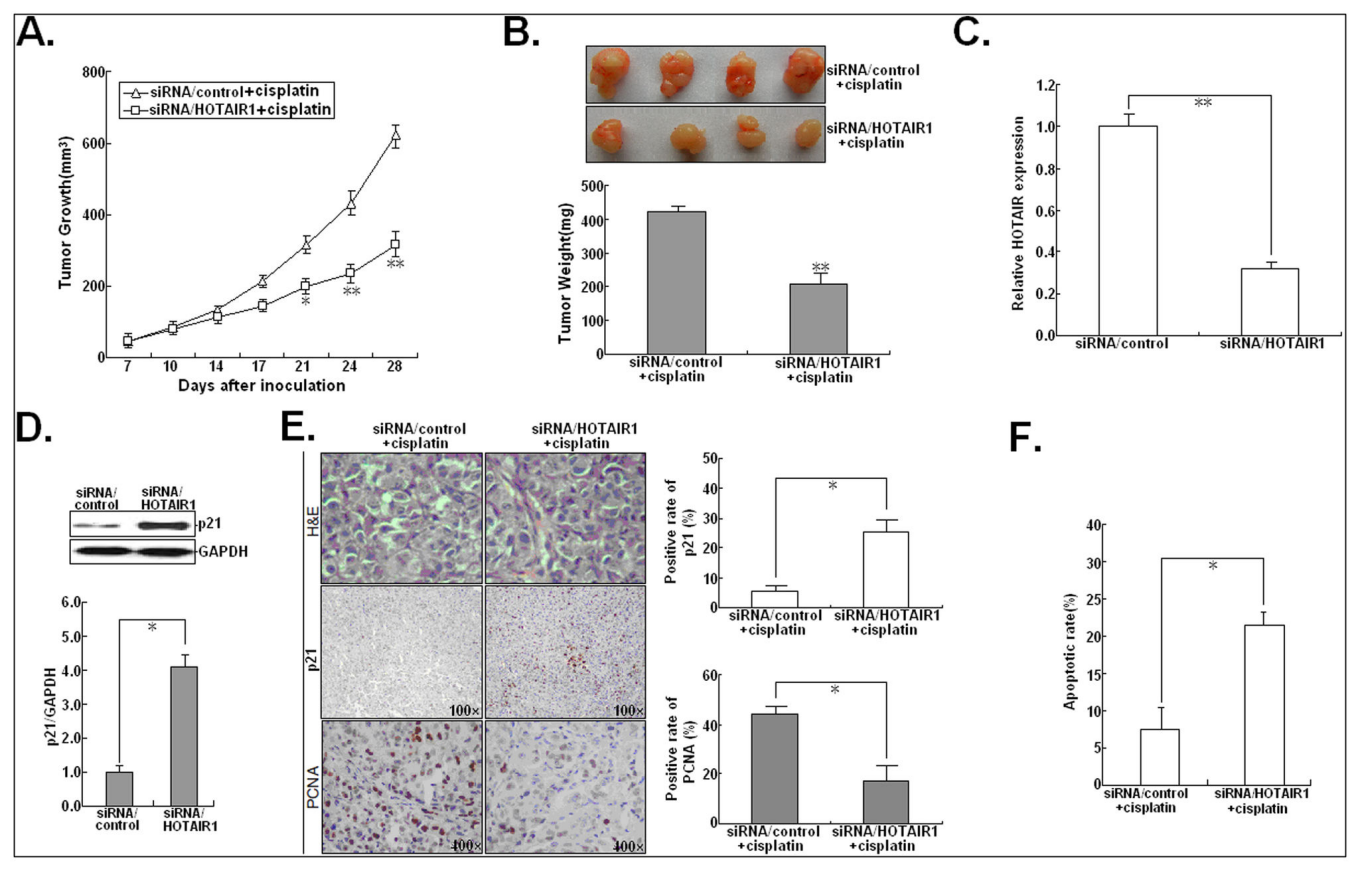
Effects of HOTAIR expression on the in vivo sensitivity of A549/DDP cells to cisplatin. (**A**) Tumor volume was calculated twice weekly following injection of A549/DDP cells transfected with siRNA/control or siRNA/HOTAIR after cisplatin treatment. Points, mean (*n*=3); bars indicate SD. (**B**) After 28 days, tumor weights are represented as means of tumor weights ± SD. (**C**) qRT-PCR detection of HOTAIR expression in tumor tissues formed from siRNA/control or siRNA/HOTAIR-transfected A549/DDP cells combined with cisplatin treatment. GAPDH was used as an internal control. (**D**) Western blot detection of p21 protein expression in tumor tissues formed from siRNA/control or siRNA/HOTAIR-transfected A549/DDP cells combined with cisplatin treatment. GAPDH was used as an internal control. (**E**) Tumors developed from siRNA/HOTAIR-transfected A549/DDP cells showed higher positive rate of p21 protein and lower positive rate of PCNA protein levels than tumors developed from siRNA/control-A549/DDP cells. Upper: H&E staining; Intermediate and lower: immunostaining, Origninal magniﬁcation, 100× or 400×. (**F**) Detection of apoptosis in tumors developed from siRNA/HOTAIR or siRNA/control-transfected A549/DDP cells combined with cisplatin treatment. Results represent the average of three independent experiments (mean±SD). * or ** indicates *P*<0.05 or <0.01, respectively.

To validate the effect of HOTAIR on the sensitivity of A549 cells to cisplatin *in vivo*, A549/DDP cells transfected with siRNA/control or siRNA/HOTAIR1 were injected into a nude mouse xenograft model. When the average tumor size reached about 50 mm^3^, this model was subsequently treated with cisplatin. As shown in Figure 6A, the tumors formed from siRNA/HOTAIR1-transfected A549/DDP cells grew significantly slower than those formed from siRNA/control-tansfected cells. All mice were killed at day 28 after the initial cisplatin administration, and the average tumor weight of A549/DDP tumor xenografts were recorded. As shown in Figure 6B, following the treatment with cisplatin, the average weight of tumors formed from siRNA/HOTAIR1 or siRNA/control-transfected A549/DDP cells was 204.8±6.8 mg and 423.4±8.4 mg, respectively, and thus, the downregulation of HOTAIR expression led to a 51.8% inhibition of tumor growth (*P*<0.01). Next, qRT-PCR and Western blot assays were performed to detect the expression of HOTAIR and p21 protein in selected tumor tissues. The expression level of HOTAIR in tumor tissues from siRNA/HOTAIR-transfected A549/DDP cells was significantly lower than that in tumor tissues from siRNA/control-transfected cells, while the expression of p21 protein in tumor tissues from siRNA/HOTAIR1-transfected cells were higher than that in tumor tissues from siRNA/control-transfected cells (*P*<0.05; Figure 6C). Likewise, immunostaining indicated that the positive rate of p21 protein in tumor tissues from siRNA/HOTAIR-transfected cells (25.4±4.2%) was significantly stronger than that in tumor tissues from siRNA/control-transfected cells (6.2±2.4%; *P*<0.05; Figure 6D). Following the treatment with cisplatin, immunostaining showed that PCNA-positive cells in tumors formed from siRNA/HOTAIR1-transfected cells (17.4±3.2%) were significantly decreased compared with that in tumors formed from siRNA/control-transfected cells (44.6±5.4%; *P*<0.05; Figure 6D), and TUNEL staining assay indicated that the rate of apoptotic tumor cells was significantly increased in tumors formed from siRNA/HOTAIR1-transfected cells compared with that in tumors formed from siRNA/control-transfected cells (*P*<0.05; Figure 6E). Therefore, upregulation of HOTAIR could decrease the in vivo sensitivity of LAD cells to cisplatin by downregulating p21 expression.

### HOTAIR-p21 dysregulation in LAD tissues is correlated with the clinical response of patients to cisplatin-based chemotherapy

**Figure 7:**
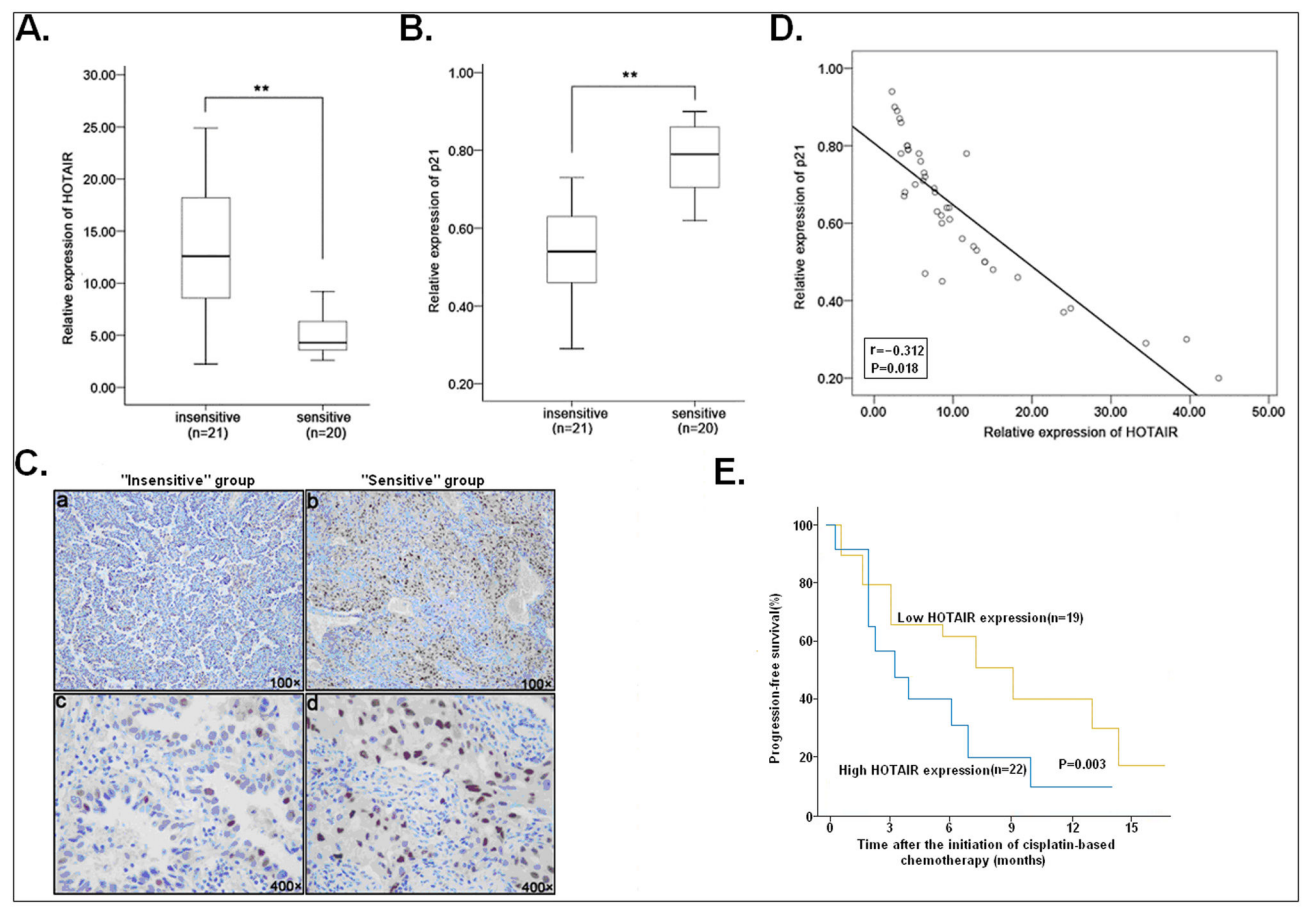
The inverse correlation between HOTAIR and p21 expression in LAD tissue samples. (**A**) qRT-PCR detection of HOTAIR expression in cisplatin-sensitive (n=21) or insensitve (n=20) LAD tissues. Abundance of HOTAIR was normalized to GAPDH. (**B**) qRT-PCR detection of p21 mRNA expression in cisplatin-sensitive (n=21) or insensitve (n=20) LAD tissues. Abundance of p21 mRNA was normalized to GAPDH. (**C**) Immunostaining of p21 protein expression cisplatin-sensitive (n=20) or insensitve (n=21) LAD tissues. Origninal magniﬁcation, 100×(upper), 400×(low). (**D**) The inversely correlated expression of HOTAIR and p21 mRNA among those LAD tissue samples (n=41) as indicated by linear regression analysis. Spearman rank test r and *P* values (2-tailed) were shown. (**E**) Kaplan-Meier survival analysis of the association between PFS of patients and HOTAIR expression according to the level of HOTAIR expression. The *P*-value was determined with the log-rank test. ** indicates *P*<0.01.

To better understand the correlation of HOTAIR-p21 dysregulation in LAD tissues and the clinical response to cisplatin-based regimens, qRT-PCR was performed to detect the relative expression level of HOTAIR and p21 mRNA on tumor tissues from 41 eligible patients with advanced LAD treated with cisplatin-based chemotherapy was performed. According to the patient’s response to cisplatin-based chemotherapy, patients were divided into ‘‘sensitive’’ (CR+PCR) and ‘‘insensitive’’ (SD+PD) groups. As shown in Figure 7A, the relative level of HOTAIR expression in the ‘‘insensitive’’ group tissues (n=21) was significantly higher than that in the ‘‘sensitive’’ group ones (n=20; *P*<0.01), suggesting that HOTAIR expression showed a strong negative correlation with the responses of patients to cisplatin-based chemotherapy (Spearman rank test rho =-0.82; *P*<0.01). On the contrary, the relative level of p21 mRNA expression in the ‘‘insensitive’’ group tissues was significantly lower than that in the ‘‘sensitive’’ group ones (*P*<0.01; Figure 7B), suggesting that HOTAIR expression showed a strong positive correlation with the responses of patients to cisplatin-based chemotherapy (Spearman rank test rho = 0.87; *P*<0.01). Further immunostaining of p21 protein in those tumor tissues indicated that 16 of 21 ‘‘insensitive’’ group tissues showed weakly positive and 14 of 20 ‘‘sensitive’’ group tissues showed strongly positive immunostaining of p21 protein (Figure 7C). Thus, the expression of p21 protein in ‘‘sensitive’’ group tissues was also upregulated compared with that in ‘‘insensitive’’ group tissues. By linear regression analysis of the correlation between HOTAIR and p21 mRNA expression in 41 tumor tissues, it was observed that there was an inverse correlation between HOTAIR and p21 mRNA in LAD tissues (Spearman rank test r = -0.312; *P* = 0.018) (Figure 7D). Then, ROC curve analysis was performed to establish the optimal cutoff value for the HSCORE of HOTAIR expression level in 41 LAD tissues, which yielded a value of 74.2 (data not shown). Kaplan-Meier survival analysis was performed to analyze the association between disease progression-free survival (PFS) of LAD patients and HOTAIR expression. As shown in Figure 7E, LAD patients with low HOTAIR expression (HSCORE<74.2; n=19) had a shorter progression-free survival (PFS) than those with high HOTAIR (HSCORE≥74.2; n=22) (*P*=0.003). These results indicated that the expression of HOTAIR in advanced LAD might be negatively correlated with the response of patients to cisplatin-based chemotherapy.

## Discussion

Recent improvements in high-throughput gene expression analysis have led to the discovery that <2% of the total human genome can be transcribed, yielding many short or long noncoding RNAs (lncRNAs) with limited or no protein-coding capacity [18,19]. One of the emerging themes in ncRNA research involves microRNAs, a class of small regulatory RNAs that mediate post-transcriptional silencing of specific target mRNAs [20]. Similarly, other classes of newly discovered lncRNAs, such as XIST and HOTAIR, have also been characterized [21]. The participation of lncRNAs in a wide repertoire of biological processes has been a topic of intense contemporary research, as virtually every step in the life cycle of genes from transcription to mRNA splicing, RNA decay, and translation can be influenced by these molecules [22-24]. Several studies have further demonstrated that lncRNAs can control gene expression by directly recruiting histone-modifying enzymes to chromatin [25]. Dysregulation of these lncRNAs may also affect epigenetic information and provide a cellular growth advantage, resulting in progressive and uncontrolled tumor growth [26]. Therefore, the interplay between proteins and lncRNAs is an important topic in the field of cancer biology, in which lncRNAs may provide the missing piece of the well-known oncogenic and tumor suppressor network puzzle.

Up to now, many lncRNAs have been identified, and their involvement in human cancer has been extensively reported. Long ncRNAs, such as lincRNA-p21, uc.73, and uc.338, have been reported to be correlated with human malignancies [27]. However, the detailed function and the clinical significance of the long ncRNAs have not yet been elucidated. The lncRNA HOTAIR was initially identified as one of the 231 lncRNAs associated with the human HOX loci; however, transcription was repressed in the distal HOXD locus of foreskin fibroblasts [28]. Recent studies of the individual functionalities of long non-coding RNAs (lncRNAs) in the development and progression of cancer have suggested that HOTAIR is capable of reprogramming chromatin organization and promoting cancer cell metastasis [29]. Gupta et al. reported that HOTAIR was highly expressed in breast cancer metastases and in primary tumors predisposed to future metastases [30]. Notably, upregulation of HOTAIR expression has been shown to be successful in targeting polycomb repressive complex 2 (PRC2), a complex comprised of histone H3-lysine 27-methylase, EZH2, SUZ12, and EED. This effect is genome-wide, serving to alter H3K27 methylation and gene expression patterns, thus increasing cancer invasiveness and metastasis *in vivo*. Also, enforced HOTAIR expression has been found in other tumors, including pancreatic cancer, colorectal cancer, hepatocellular carcinoma, and gastrointestinal stromal tumors [12-14,31]. However, the correlation of HOTAIR with chemosensitivity of tumor cells is unclear and remains to be elucidated. 

Tumor chemoresistance remains one of the most significant challenges to successful treatment of lung cancer [32]. Currently, majority of cancer patients evidencing initial responsiveness to treatment will develop aggressive malignancies. Such malignant cells may exhibit up to 90% resistance to one or more drugs. Despite its clinical prevalence, the underlying mechanisms of resistance to chemotherapeutic agents are still poorly understood. Although evidence regarding genetic alteration following chemotherapeutic treatment is limited, numerous studies have demonstrated substantial epigenetic alterations in drug-resistant cancer cells [33]. The lncRNA HOTAIR has been previously shown to bind PRC2 or LSD1/CoREST/REST, mediate H3K27 trimethylation and H3K4 demethylation, and regulate global gene expression. This is the first report to investigate the correlation between HOTAIR expression and tumor chemoresistance. Here, the expression of HOTAIR was found to be significantly upregulated in A549/DDP cells compared with parental A549 cells, and the expression of HOTAIR in parental A549 cells decreased gradually according to the cisplatin treatment. Then, we attempted to investigate the roles of HOTAIR in cisplatin resistance of LAD cells and its possible molecular mechanisms by using gain- or loss-of-function approaches. In our studies, siRNA-mediated downregulation of HOTAIR could reverse the resistance of chemoresistant A549/DDP cells to cisplatin, while upregulation of HOTAIR could significantly decrease the *in vitro* sensitivity of parental A549 and SPC-A1 cells to cisplatin. Also, downregulation of HOTAIR could increase the *in vivo* sensitivity of LAD cells to cisplatin. Further researches indicated that the mechanisms of siRNA/HOTAIR1-increased chemosensitivity of LAD cells to cisplatin might be associated with apoptosis enhancement and G_0_/G_1_ cell cycle arrest. In previous studies, HOTAIR is reported to work in cooperation with the PRC2 and LSD1/CoREST/REST, which results in the modifications of DNA-binding proteins and then regulates global gene expression. EZH2 and SUZ12, the components of PRC2, have been found to overexpress in a lot of human cancers, including lung cancer [34,35]. EZH2 protein in cancer cells has been found to be closely associated with p21waf1/cip1, pointing to EZH2 as being involved with p21waf1/cip1 regulation [36,37]. Therefore, we explored whether p21 might be an important mediator for HOTAIR-induced cisplatin resistance of LAD cells. In this study, siRNA/HOTAIR1 could significantly induce the increased expression of p21 protein in A549/DDP cells, while pcDNA/HOTAIR could significantly decrease the expression of p21 protein in A549 and SPC-A1 cells. These data implied that p21 was a downstream target of HOTAIR. Functional analyses showed that overexpression of p21 could mimic the effect of siRNA/HOTAIR1 on the sensitivity of A549/DDP cells to cisplatin and downregulation of p21 could mimic the effect of pcDNA/HOTAIR on the sensitivity of A549 cells to cisplatin. More importantly, siRNA/p21 or pcDNA/p21 could partially reverse the effects of siRNA/HOTAIR1 or pcDNA/HOTAIR on not only the expression of p21 protein but also the cisplatin sensitivity in cisplatin-resistant or parental LAD cells. Taken together, these results suggested that HOTAIR could promote the cisplatin resistance of LAD cells by downregulating p21 expression. However, the molecular mechanisms involved in HOTAIR-induced p21 downregulation need to be further elucidated in future. 

p21WAF1 (p21) is a cyclin-dependent kinase inhibitor that is induced by p53 upon DNA damage or p53 overexpression, resulting in cell cycle arrestat the G1 checkpoint and inhibition of further cell proliferation [38]. Previously, p21 was found to be low-expressed in lung cancer tissues and the group of patients whose lung cancer specimens were negative for p21 had significantly shorter overall survival [39]. In addition, p21WAF1 adenoviral gene transfer has been reported to lead to the inhibition of lung cancer cell growth by inducing G_0_/G_1_ arrest [40]. Also, p21 is reported to function as a tumor suppressor in other human cancers, including pancreatic cancer, breast cancer, hepatocelluar carcinoma, and so on [41]. Epigenetic silencing is a common mechanism to inactivate tumor suppressor genes during carcinogenesis. Enhancer of Zeste 2 (EZH2) is the histone methyltransferase subunit in polycomb repressive complex 2 which mediates transcriptional repression through histone methylation [42]. Cao and colleagues have reported that p21 could be significantly increased in non-small cell lung cancer cells after EZH2-siRNA delivery [36]. Meanwhile, the expression of p21 in human tumors could be post-transcriptionally regulated by a lot of microRNAs including miR-663 and miR-423 [43,44]. However, the effects of lncRNAs on the regulation of p21 expression are not reported. To further testify the correlation between dysregulation of HOTAIR/p21 and the sensitivity of LAD cells to cisplatin, we analyzed the expression of HOTAIR and p21 mRNA or protein in “cisplatin-sensitive” or “cisplatin-insensitive” LAD tissues, and found that the expression of HOTAIR in LAD tissues was negatively correlated with the responses of LAD patients to cisplatin-based chemotherapy. However, the expression of p21 in LAD tissues was positively correlated with the responses of patients to chemotherapy, and p21 mRNA showed an inverse correlation with HOTAIR, which was also detected in nude mice A549/DDP tumor xenografts treated with siRNA/HOTAIR1. 

In conclusion, the current study provided novel indications that lncRNAs, specifically HOTAIR, can regulate the cisplatin-resistance ability of human LAD cells through regulation of apoptosis and cell cycle distribution by affecting p21 expression. This study is an important step towards development of a complete understanding of the pathogenesis and development of the cisplatin resistance in human LAD. This raises the possibility that anti-HOTAIR may have potential therapeutic value for those cisplatin-resistant LAD patients. While the goal of this study was to better understand HOTAIR function in the cisplatin resistance of LAD cells, future research is required to address the therapeutic potential of modulating HOTAIR.

## Supporting Information

Figure S1 **pcDNA/HOTAIR significantly promotes the resistance of SPC-A1 cells to cisplatin.** (**A**) qRT-PCR detection of HOTAIR expression in SPC-A1cells stably transfected with pcDNA/control or pcDNA/HOTAIR. GAPDH was used as an internal control. (**B**) MTT analysis of the IC_50_ values of cisplatin to SPC-A1/control or SPC-A1/HOTAIR cells. (**C**) Flow cytometry analysis of apoptosis in SPC-A1/control or SPC-A1/HOTAIR cells combined with various concentrations of cisplatin (0.0, 1.0 or 1.5 μg/L). (**D**) Flow cytometry analysis of cell cycle distribution in SPC-A1/control or SPC-A1/HOTAIR cells combined with various concentrations of cisplatin (0.0, 1.0 or 1.5 μg/L). Results represent the average of three independent experiments (mean±SD). N.S indicates *P*>0.05 and * or ** indicates *P*<0.05 or <0.01, respectively.
(TIF)

Figure S2 **pcDNA/21 reverses the effects of pcDNA/HOTAIR on the chemosensitivity of SPC-A1 cells to cisplatin.** (**A**) Western blot analysis of the effect of HOTAIR on p21 protein expression in pcDNA/HOTAIR (or pcDNA/control) or siRNA/HOTAIR1 (or siRNA/control)-transfected SPC-A1 cells. GAPDH was used as an internal control. (**B**) 48h after SPC-A1 cells transfected with pcDNA/control, pcDNA/HOTAIR alone or combination with pcDNA/p21, Western blot detection of p21 protein expression in those cells. GAPDH was used as an internal control. (**C**) MTT analysis of the IC_50_ values of cisplatin to SPC-A1 cells transfected with pcDNA/control, pcDNA/HOTAIR alone or combination with pcDNA/p21. Results represent the average of three independent experiments (mean±SD). *N.S* indicates *P*>0.05 and * or ** indicates *P*<0.05 or <0.01, respectively.
(TIF)
