# Supplementary figures and images for: The Long Noncoding RNA HOTAIR Contributes to Cisplatin Resistance of Human Lung Adenocarcinoma Cells via downregualtion of p21WAF1/CIP1 Expression
Source: PLoS One. 2013 Oct 14;8(10):e77293. doi: 10.1371/journal.pone.0077293 (PMC3796503; doi:10.1371/journal.pone.0077293)

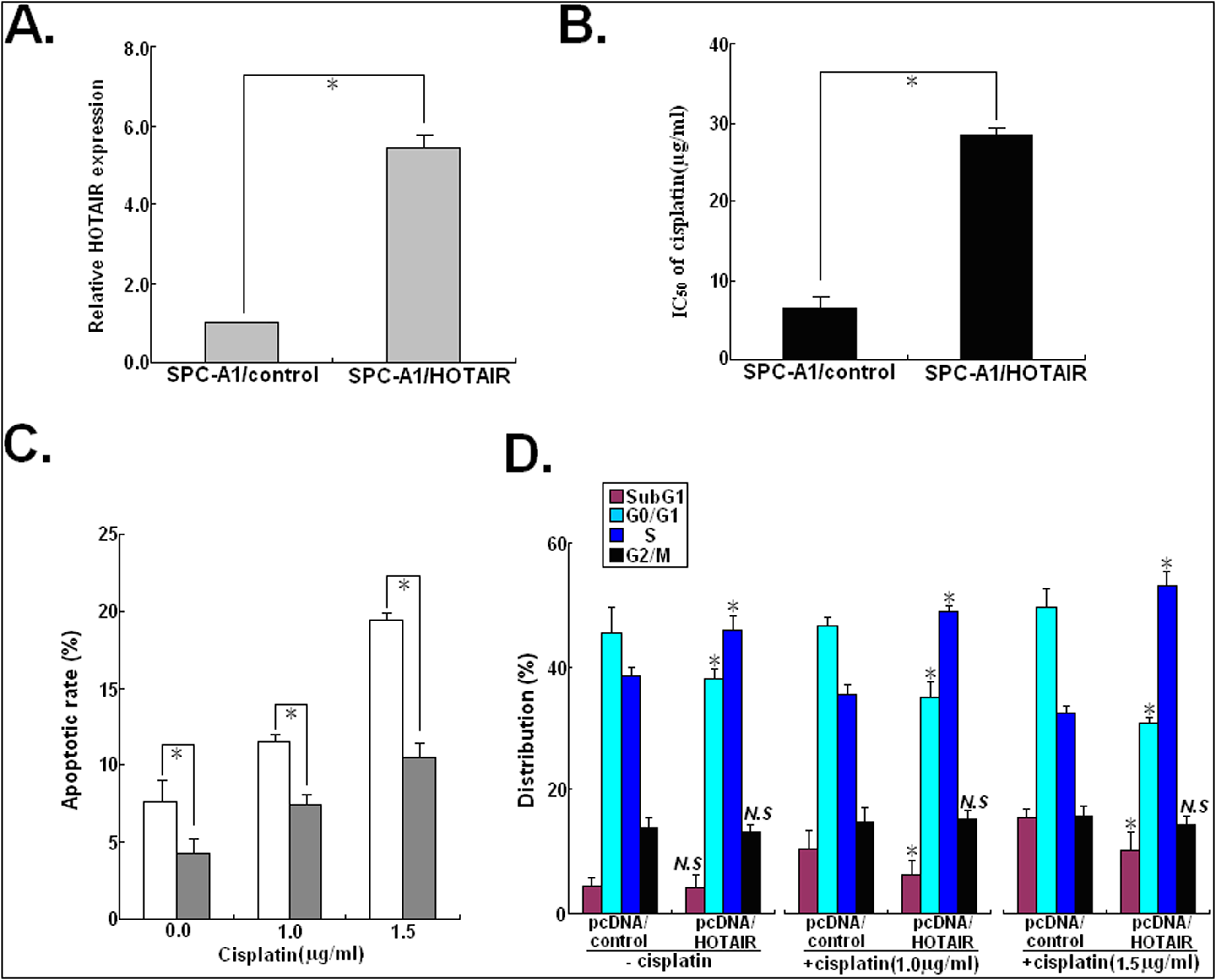

Supplement: Figure S1 — pcDNA/HOTAIR significantly promotes the resistance of SPC-A1 cells to cisplatin. (A) qRT-PCR detection of HOTAIR expression in SPC-A1cells stably transfected with pcDNA/control or pcDNA/HOTAIR. GAPDH was used as an internal control. (B) MTT analysis of the IC50 values of cisplatin to SPC-A1/control or SPC-A1/HOTAIR cells. (C) Flow cytometry analysis of apoptosis in SPC-A1/control or SPC-A1/HOTAIR cells combined with various concentrations of cisplatin (0.0, 1.0 or 1.5 μg/L). (D) Flow cytometry analysis of cell cycle distribution in SPC-A1/control or SPC-A1/HOTAIR cells combined with various concentrations of cisplatin (0.0, 1.0 or 1.5 μg/L). Results represent the average of three independent experiments (mean±SD). N.S indicates P>0.05 and * or ** indicates P<0.05 or <0.01, respectively. (TIF) [file pone.0077293.s001.tif]

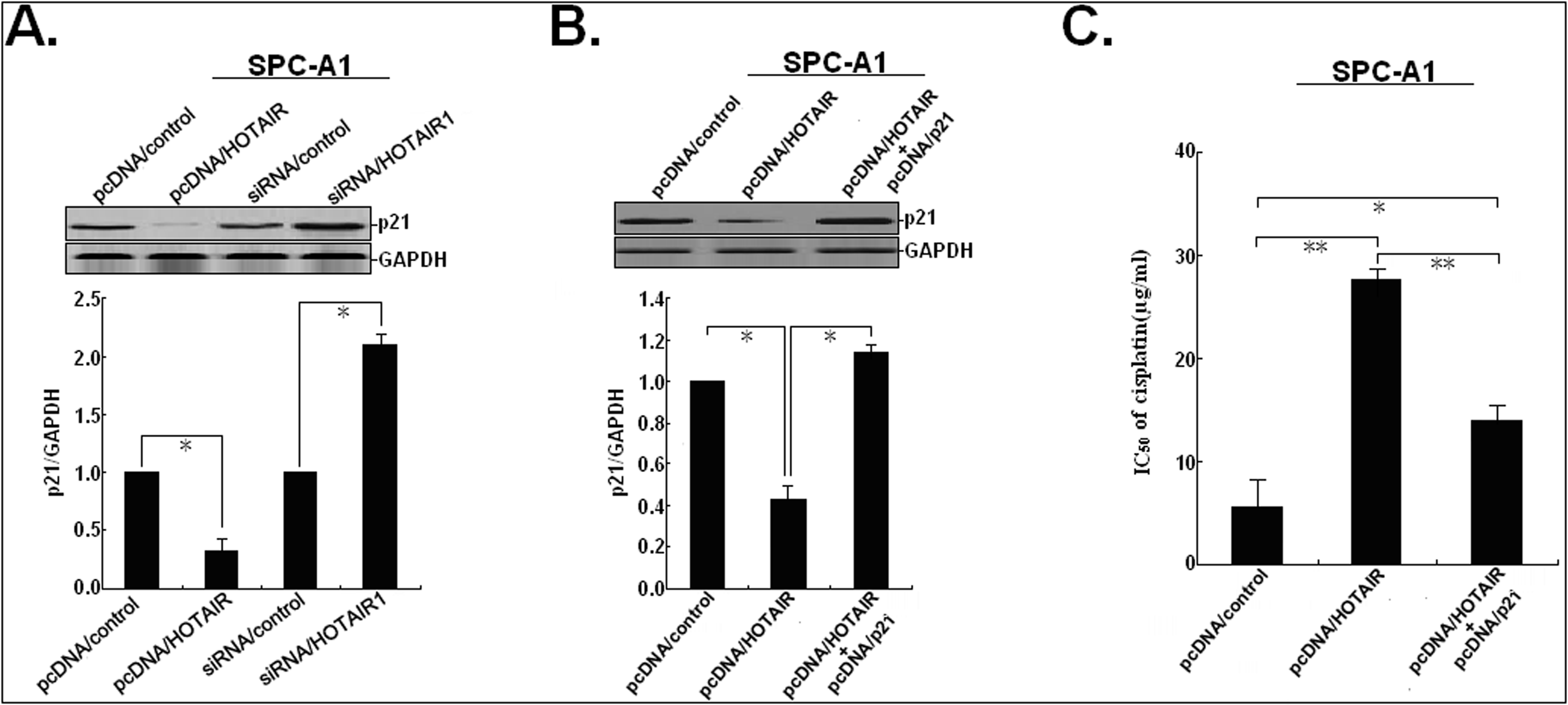

Supplement: Figure S2 — pcDNA/21 reverses the effects of pcDNA/HOTAIR on the chemosensitivity of SPC-A1 cells to cisplatin. (A) Western blot analysis of the effect of HOTAIR on p21 protein expression in pcDNA/HOTAIR (or pcDNA/control) or siRNA/HOTAIR1 (or siRNA/control)-transfected SPC-A1 cells. GAPDH was used as an internal control. (B) 48h after SPC-A1 cells transfected with pcDNA/control, pcDNA/HOTAIR alone or combination with pcDNA/p21, Western blot detection of p21 protein expression in those cells. GAPDH was used as an internal control. (C) MTT analysis of the IC50 values of cisplatin to SPC-A1 cells transfected with pcDNA/control, pcDNA/HOTAIR alone or combination with pcDNA/p21. Results represent the average of three independent experiments (mean±SD). N.S indicates P>0.05 and * or ** indicates P<0.05 or <0.01, respectively. (TIF) [file pone.0077293.s002.tif]
